# Determination of the Optimum Conditions for Emulsification and Encapsulation of Echium Oil by Response Surface Methodology

**DOI:** 10.1021/acsomega.3c01779

**Published:** 2023-07-28

**Authors:** Hamdy A. Zahran, Gizem Catalkaya, Hande Yenipazar, Esra Capanoglu, Neşe Şahin-Yeşilçubuk

**Affiliations:** a Fats and Oils Department, Food Industries and Nutrition Research Institute, National Research Centre, Dokki, Cairo, 12622, Egypt; b Department of Food Engineering, Faculty of Chemical and Metallurgical Engineering, Istanbul Technical University, Maslak, Istanbul 34469, Turkey

## Abstract

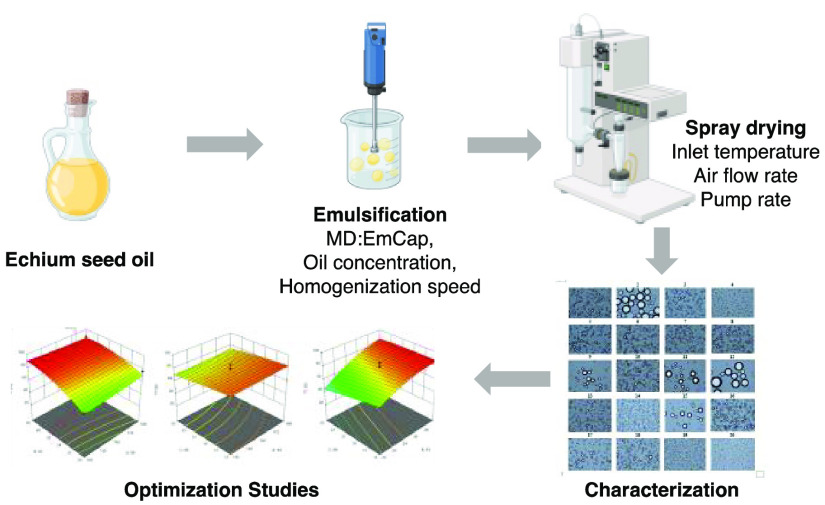

Echium oil (EO) contains substantial amounts of omega-3 fatty acids, which are important because of their benefits to human health. However, they are prone to oxidation. The aim of this study was to obtain the optimum conditions of microencapsulation of EO using spray drying by applying the response surface methodology (RSM). Central composite circumscribed design (CCC) was employed with a ratio of maltodextrin (MD):EmCap modified starch (MS) (80–90%, w/w), oil concentration (15–25%, w/w), and homogenization speed (5–15 × 10^3^ rpm) as independent variables affecting droplet size (μm) and viscosity (Pa·s), which were chosen as responses for the emulsification process. The results revealed that the emulsion conditions containing MD:MS (89.7%:10.3%, w/w), oil concentration of (16.0%), and homogenization speed at (14.8 × 10^3^ rpm) were found to be the optimum conditions. Furthermore, for encapsulation, CCC was employed with inlet temperature of 140–180 °C, air flow of 20–30%, and pump rates of 15–25% as independent variables. Total yield (%) and encapsulation efficiency (%) were chosen as responses for the encapsulation process. On the other hand, optimum conditions for encapsulation were as follows: inlet temperature of 140 °C, airflow rate of (30%) 0.439 m^3^/h, pump rate of (15%) 4.5 mL/min with respect to selected responses.

## 1 Introduction

The knowledge about the beneficial effects of polyunsaturated fatty acids (PUFA) on inflammatory diseases such as cancer, asthma and allergic diseases has increased significantly in recent years.^1^ The most important fatty acids in PUFA are omega-3 and omega-6 fatty acids. However, nowadays people have started to search for new omega-3 sources, because fish oil stocks, which are known as a good omega-3 source, are decreasing day by day and animal based omega-3 sources are not preferred by some consumers.^2^ Echium oil (EO) is extracted from the seeds of *Echium plantagineum* and it is one of the vegetable oils containing omega-3 and omega-6 fatty acids.^3,4^ It contains significant amounts of PUFAs like linoleic acid (LA, 18:2n-6) (19%), γ-linolenic acid (GLA, 18:3n-6) (10%), α-linolenic acid (ALA, 18:3n-3) (30%) and stearidonic acid (SDA, 18:4n-3) (13%).^1,5^ In addition, SDA is an intermediate metabolite in the synthesis of eicosapentaenoic acid (EPA) and docosahexaenoic acid (DHA).^6,7^ with a higher conversion efficiency than ALA, which enables Echium oil to be a sustainable EPA source.^8,9^

PUFAs are susceptible to oxidation and readily produce hydroperoxides, off flavours and odors, which are deemed undesirable by consumers.^10^ Thus, oxidation results in diminished nutritional and sensory properties of PUFA containing foods.^11^ Microencapsulation technology is used for the protection of unsaturated fatty acids against oxidation and other unwanted reactions. The microencapsulation process involves coating or entrapping a sensitive core material using a biopolymer material(s) in order to protect the material against adverse environmental conditions, thereby increasing the shelf life and promoting the controlled release of the active compound in the microcapsule.^12,13^ Omega-3 and omega-6 fatty acids or oils containing these PUFAs have been microencapsulated by using different encapsulation techniques. Currently, spray drying dominates the industrial microencapsulation of omega-3 and -6 PUFAs. Spray drying is a fast, continuous, and high temperature technique.^14^ It offers many advantages over other drying methods including an ability to handle heat-sensitive materials, low operational cost, readily available machinery, and reliable operation and the ability to control the mean particle size of the powders for spray dried emulsions.^10,13^

Different coating materials are used for encapsulation purposes, such as proteins, carbohydrates, and lipids, etc. Coating materials should have barrier properties against environmental conditions, which are light, oxygen, moisture, etc., and desired release characteristics of the encapsulated ingredient.^15^ Generally, materials having different characteristics are combined together to achieve the quality of interest.^16^ Among the polysaccharides, starches and starch derivatives are widely used wall materials.^17^ Modified starch (MS) is a starch derivative obtained by physical, chemical, or enzymatic modifications of native starch in the aim of enhancing the properties for encapsulation applications.^18^ Maltodextrin (MD) is another type of starch hydrolysate that provides oxidative stability of the core material. Due to this property, it is a good alternative for the encapsulation of lipids.^19^

Response surface methodology (RSM), which combines mathematics with statistics, has been applied successfully in food processing operations.^20^ Analyzing the effects of the independent variables, this methodology generates a mathematical model which describes the chemical processes within the experimental range.^21^ RSM has also been used by some authors to efficiently optimize the parameters of the encapsulation process by spray drying.^21−23^

In previous studies, gelatin-Arabic gum,^3^ gelatin-cashew gum^12^ and sodium alginate^24^ were used as wall material in EO encapsulation. To the best of our knowledge, there has been no previous study in which modified starch and maltodextrin were used as wall materials in the EO encapsulation process. The combination of maltodextrin and modified starch was chosen in our study to improve the functionality and stability of the final product. Maltodextrin has good solubility and contributes to the mouthfeel and texture of the product, while modified starch enhances its viscosity, stability, and resistance to shear and heat. Additionally, this combination allows for the reduction of the total amount of starch used, which can lead to cost savings and improved nutritional value.^25,26^ In addition, it was observed that the freeze-drying method was generally used in the studies examined.

This study investigated the optimum conditions for the microencapsulation of EO by using RSM. Wall material ratio, oil concentration, and homogenization rate were the factors affecting the emulsification of EO. Also, during spray drying optimization, inlet temperature, air flow rate, and pump speed were changed to optimize oil retention and reduce surface oil content in the encapsulated material. In this way, emulsion and spray drying characteristics of EO were investigated for the first time to obtain a value-added material potentially to be used as an ingredient in food, pharmaceutical, and cosmetical products.

## 2 Results and Discussion

### 2.1 Quality Properties and Fatty Acid Composition of EO

According to the test results, the free fatty acid content (% as oleic acid) and peroxide value (PV) of the EO were found to be 0.38% and 0.85 mEquiv of O_2_/kg, respectively. These values were found to be at acceptable levels set by the Codex Alimentarius Commission.^27^

The fatty acid profile of EO is given in Table 1. It was shown that the unsaturated fatty acid (UFA) level was high 87.89%. The α-linolenic acid (ALA, 18:3 n3) was the most abundant UFA in EO (33.03%). The percentage of omega-3 fatty acids reached 46.52%, whereas omega-6 and omega-9 fatty acid contents were found to be 24.83% and 16.55%, respectively. Castejon et al.^28^ reported that *Echium plantagineum* L. seeds can contain omega-3 fatty acids up to 50.25 to 51.73%.

**Table 1:** Fatty Acid Composition of EO

Fatty Acids^a^	Area (%)
Palmitic acid (16:0)	6.85 ± 0.23
Stearic acid (18:0)	3.90 ± 0.02
Oleic acid (18:1) n9, cis	15.41 ± 0.07
Elaidic acid (18:1) n9, trans	0.71 ± 0.01
Linoleic acid (18:2) n6	14.43 ± 0.06
α-Linolenic acid (18:3) n3	33.03 ± 0.15
γ-Linolenic acid (18:3) n6	10.41 ± 0.05
Stearidonic acid (C18:4) n3	13.49 ± 0.11
Arachidic acid (20:0)	0.89 ± 0.06
Gondoic acid (20:1)	0.43 ± 0.01
Behenic acid (22:0)	0.21 ± 0.01
*Others*	0.27
% SFA	11.84
% UFA	87.89
% ω-3 Fas	46.52
% ω-6 Fas	24.83
% ω-9 Fas	16.55

a EO, Echium Oil; SFA, Saturated fatty acids; UFA, Unsaturated fatty acids.

Castejon et al.^28^ extracted *Echium plantagineum* L. seed oils by pressurized liquid extraction (PLE), microwave assisted extraction (MAE), and ultrasound assisted extraction (UAE). The content of alpha-linolenic acid was found to be in the range of 35.31%–36.16%, and the rate of ALA obtained by the traditional method was similar to our results (35.31% ± 0.25).

Also in another study,^20^ researchers extracted *Echium vulgare* seed oil using supercritical carbon dioxide extraction method. They found that the UFA and ALA contents of *Echium vulgare* seed oil were 88.5 ± 0.2 and 33.5 ± 0.1, respectively.

It can be concluded that the total UFA and ALA content of *Echium plantagineum* seed oil was similar to *Echium vulgare* seed oil and extraction methods may cause minor differences in fatty acid contents of EO.

### 2.2 Emulsion Optimization and Characterization

Three factors were chosen for emulsion preparation: ratio of wall materials (MD:MS) (*X*_1_,%) (80–90% w/w), oil concentration (*X*_2_,%) (15–25%, w/w), and homogenization speed (*X*_3_, rpm) (5–15 × 10^3^ rpm). Based on the selected independent variables, RSM was used to investigate the effects of these factors on emulsion droplet size (μm) and viscosity (Pa·s).

**Figure 1:**
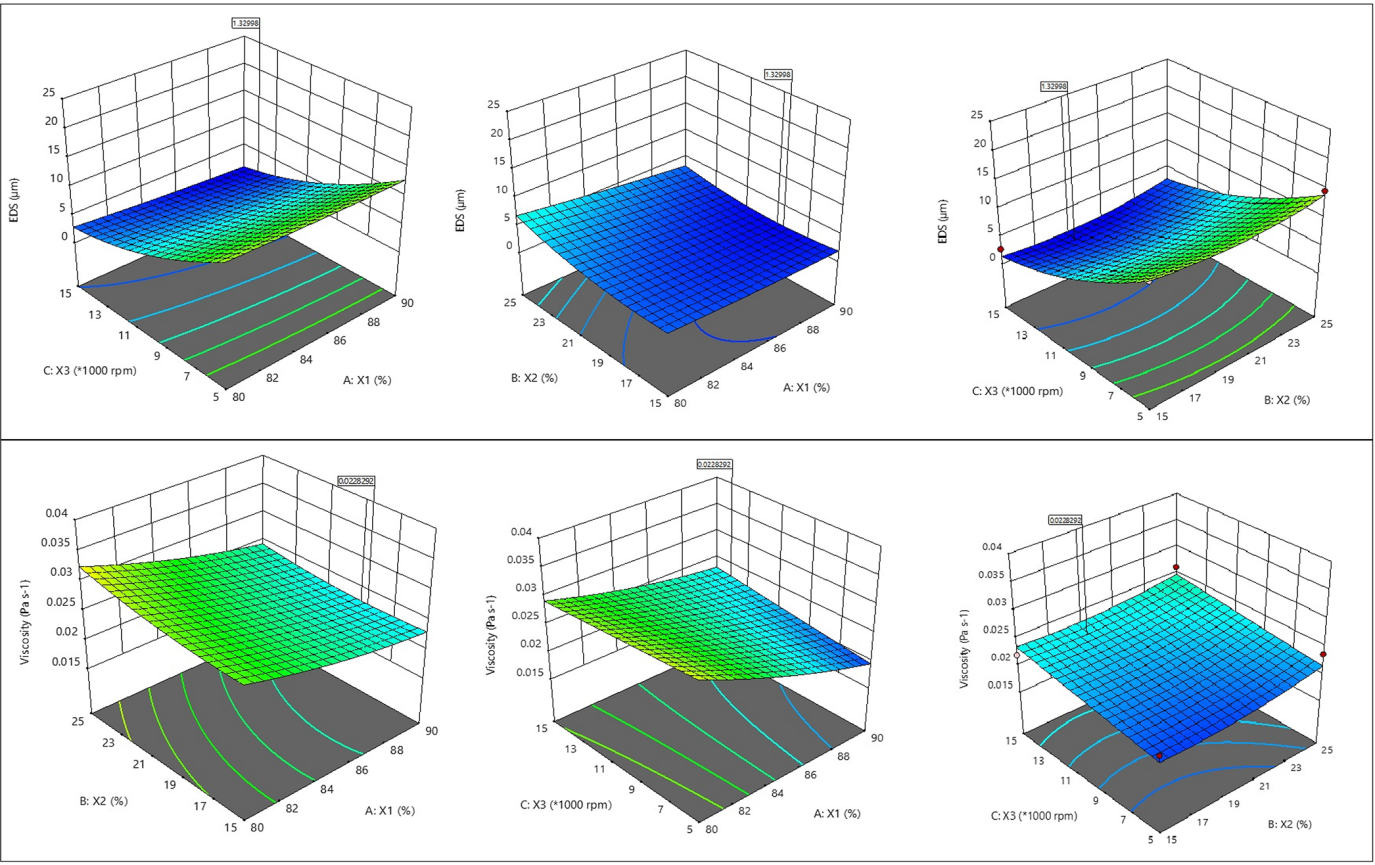
Emulsion droplet size (μm) (upper row) and viscosity (Pa·s) (lower row) plots of the sample.

Table 2 shows the central composite design for the optimization of emulsion preparation, and Table 3 shows the observed values of dependent variables for different runs of emulsification optimization experiments. 3D response surface plot between two parameters (emulsion droplet size (μm) and viscosity (Pa·s)) for emulsification is given in Figure 1.

**Table 2:** Central Composite Design for the Optimization of Emulsion Preparation and Spray Drying Conditions^a^

	Coded variables			Emulsification variables			Coded variables			Encapsulation variables		
Run No.	*X*_1_	*X*_2_	*X*_3_	(*W*)	(*O*)	(*H*)	(*X*_4_)	(*X*_5_)	(*X*_6_)	(*T*_i_)	(*A*)	(*P*)
1	0	0	0	85.0	20.0	10.0	0	0	0	160.0	25.0	20.0
2	1	1	–1	90.0	25.0	5.0	0	0	0	160.0	25.0	20.0
3	0	2	0	85.0	28.4	10.0	2	0	0	193.6	25.0	20.0
4	1	–1	1	90.0	15.0	15.0	–1	–1	1	140.0	20.0	25.0
5	–1	1	1	80.0	25.0	15.0	–1	1	1	140.0	30.0	25.0
6	0	–2	0	85.0	11.6	10.0	0	2	0	160.0	33.4	20.0
7	0	0	0	85.0	20.0	10.0	–1	1	–1	140.0	30.0	15.0
8	0	0	0	85.0	20.0	10.0	0	0	0	160.0	25.0	20.0
9	–1	1	–1	80.0	25.0	5.0	1	–1	–1	180.0	20.0	15.0
10	–2	0	0	76.6	20.0	10.0	–1	–1	–1	140.0	20.0	15.0
11	1	–1	–1	90.0	15.0	5.0	0	0	2	160.0	25.0	28.4
12	0	0	–2	85.0	20.0	1.6	0	0	0	160.0	25.0	20.0
13	0	0	0	85.0	20.0	10.0	0	0	0	160.0	25.0	20.0
14	–1	–1	1	80.0	15.0	15.0	1	–1	1	180.0	20.0	25.0
15	–1	–1	–1	80.0	15.0	5.0	1	1	1	180.0	30.0	25.0
16	0	0	0	85.0	20.0	10.0	0	0	0	160.0	25.0	20.0
17	0	0	0	85.0	20.0	10.0	1	1	–1	180.0	30.0	15.0
18	2	0	0	93.4	20.0	10.0	–2	0	0	126.4	25.0	20.0
19	1	1	1	90.0	25.0	15.0	0	–2	0	160.0	16.6	20.0
20	0	0	2	85.0	20.0	18.4	0	0	–2	160.0	25.0	11.6

a *W*, wall materials (%, w/w); *O*, oil concentration (%, w/w); *H*, homogenization speed (*x* × 1000 rpm); *T*_i_, inlet temperature (°C); *A*, air flow rate (%), and *P*, pump rate (%).

**Table 3:** Observed Values of Dependent Variables for Different Runs of Emulsification and Encapsulation Optimization Experiments

	Emulsion		Encapsulation	
Run No.	EDS (μm)	Viscosity (Pa·s)	Total yield (%)	Encapsulation efficiency (%)
1	5.11	0.028	64.9	91.8
2	14.67	0.024	76.8	93.9
3	11.25	0.027	74.9	92.8
4	2.74	0.022	43.6	93.5
5	7.12	0.032	86.3	95.2
6	6.29	0.027	88.6	95.8
7	5.26	0.026	89.4	95.6
8	5.92	0.029	79.7	93.1
9	15.52	0.037	75.8	90.9
10	7.48	0.036	62.9	91.8
11	13.81	0.021	62.1	96.5
12	20.20	0.022	85.1	94.1
13	5.57	0.024	79.6	90.0
14	2.48	0.027	56.5	90.6
15	14.90	0.031	89.6	94.0
16	6.86	0.024	79.9	93.5
17	5.32	0.021	88.6	92.9
18	4.13	0.018	78.6	94.3
19	2.08	0.026	26.8	88.8
20	1.33	0.028	82.2	93.6
*R*^*2*^	0.98	0.80	0.91	0.75

#### 2.2.1 Emulsion Droplet Size (EDS)

**Figure 2:**
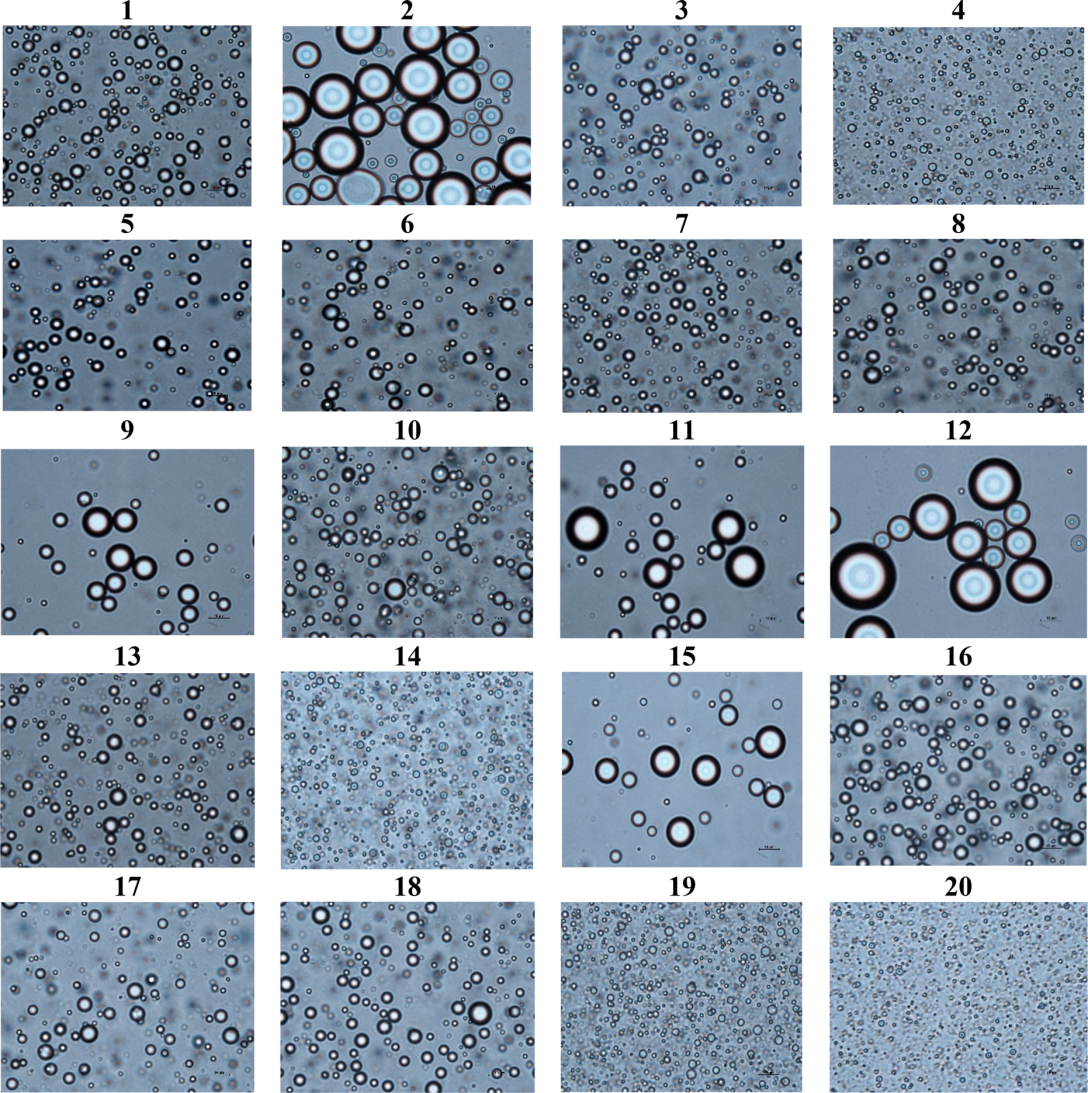
Microscopic view (×100) of prepared emulsions under different conditions (wall materials, oil concentration, and homogenization speed).

Droplet size distribution of the emulsion was affected by the ratio of the wall materials (MD:MS), oil concentration, and homogenization speed. In the emulsion preparation step, the homogenization speed was found to be the most important factor which has a significant effect (*p* < 0.05) on EDS (Figure 1). The droplet size of the prepared emulsions was observed to range from 1.33 to 20.2 μm (Table 3). In other words, by increasing the homogenization speed, the EDS was decreased. For example, the droplet size of emulsion in run no. 16 was 6.86 μm at a speed of 10 × 10^3^ rpm, however, this size decreased to 1.33 μm at the speed of 18.4 × 10^3^ rpm for run no. 20. Wu, Xiong, and Chen^29^ has also indicated that olive oil was efficiently dispersed into small droplets by increasing homogenization speed (9.5–24 × 10^3^ rpm) in the presence of Tween 80 or myofibrillar protein emulsifiers. The results of EDS measured by optical microscopy (Figure 2) indicated that EDS increases by decreasing the homogenization speed.

The goodness of fit of the model can be evaluated by determination of the coefficient (*R*^2^) value. Moreover, the significance of each coefficient was determined by using *F*-value and *p*-value.

Yolmeh et al.^30^ reported that, a high *F*-value and a small *p*-value show a more significant effect on the identical response variable. On the other hand, the model would be significant at a 95% confidence level if the *F* test has a *p*-value lower than 0.05.^30^

Determination of the coefficient value was found to be 0.983, indicating the significance of the model. According to the ANOVA analysis, the model generated also represented a satisfactory prediction since the *F* value of the model (64.192) was high (α = 0.05). Besides, there was no significant (*p* > 0.05) lack of fit in the model since the lack of fit value was 0.106.

The actual equation of the quadratic model developed from the experimental data to predict the EDS of an emulsion in terms of coded variables is given in eq 1:
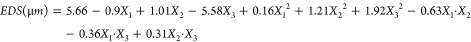
1where *X*_1_ is MD:MS ratio, *X*_2_ is oil concentration, and *X*_3_ is homogenization speed.

#### 2.2.2 Emulsion Viscosity

The emulsion viscosity was measured through steady-state shear flow curves. The Newtonian model was used to adjust the experimental data where viscosity is constant with the shear rate. All samples presented Newtonian (power-law) rheological behavior. The viscosity values of emulsions produced with the RSM design are shown in Table 3. The emulsion prepared in run no. 9 showed the highest viscosity (0.037 Pa·s), whereas the lowest viscosity was obtained in run no. 18 (0.018 Pa·s) (Figure 1). Bae and Lee,^31^ microencapsulated cold press avocado oils by spray drying in four different wall systems consisting of whey protein isolate (WPI) alone or in combination with maltodextrin (MD) DE 5 at various ratios (90:10, 50:50, and 10:90).

They observed that the viscosities were affected by the WPI-to-MD ratios despite slight differences. In our study, it can be interpreted that as the wall material ratio increases viscosity decreases. Also Carneiro et al.^32^ reported that different biopolymers and combination of different wall materials had an effect on emulsion viscosity. Based on the results, it can be concluded that different wall materials, oil concentration, and homogenization speed affect the emulsion viscosity.

The ANOVA analysis of quadratic model shows that the model is significant (*p* < 0.05) with lack-of-fit value of 0.470. Also, the actual equation of the quadratic model generated from the obtained data to predict the viscosity of emulsion in terms of coded variables is presented in eq 2: (*R*^2^ = 0.803, *F* = 4.518)
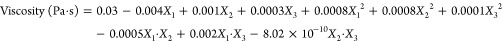
2Where, *X*_*1*_ is MD:MS ratio, *X*_*2*_ is oil concentration and *X*_*3*_ is homogenization speed.

### 2.3 Powder Optimization and Characterization

In the present study, RSM has been applied to optimize the conditions and develop a model for the encapsulation of EO by the spray drying technique. Effect of inlet temperature (*X*_4_), air flow rate (*X*_5_), and pump rate (*X*_6_) on the total yield (%) and encapsulation efficiency (EE) (%) were investigated. The central composite design for the optimization of spray drying conditions is shown in Table 2, and observed values of dependent variables for different runs of encapsulation optimization experiments are shown in Table 3. 3D response surface plot between two parameters (total yield (%) and encapsulation efficiency (%)) for emulsification is given in Figure 2.

Fitness of the model was evaluated by correlation coefficient value according to the results of ANOVA for the different characteristics of the obtained powder. The *R*^2^ values of the observed responses were 0.92 for total yield, and 0.75 for EE, indicating that the models adequately explained the relationship between the parameters chosen.

#### 2.3.1 Total Yield Percentage

Table 3 presents the characterization of powder particles prepared according to the RSM design in order to investigate the effects of spray-drying variables (inlet temperature, air flow rate, and pump rate) on the characteristics of the particles. According to the results, the samples showed significant differences (*p* < 0.05) in the yield, when different levels of parameters were used. The total yield of obtained powders (both coarse and fine) ranged from 26.8 to 89.6% (Table 3). Coefficient of determination was found to be 0.92. Moreover, ANOVA analysis revealed that the model is significant (*p* < 0.05) with a lack-of-fit value of 0.396. The actual equation of the quadratic model created from the obtained data to predict the total yield of powder during encapsulation of EO is presented in eq 3: (*R*^2^ = 0.91, lack of fit = 0.496)
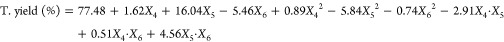
3where *X*_4_ is inlet temperature, *X*_5_ is air flow rate, and *X*_6_ is pump rate.

#### 2.3.2 Encapsulation Efficiency

**Figure 3:**
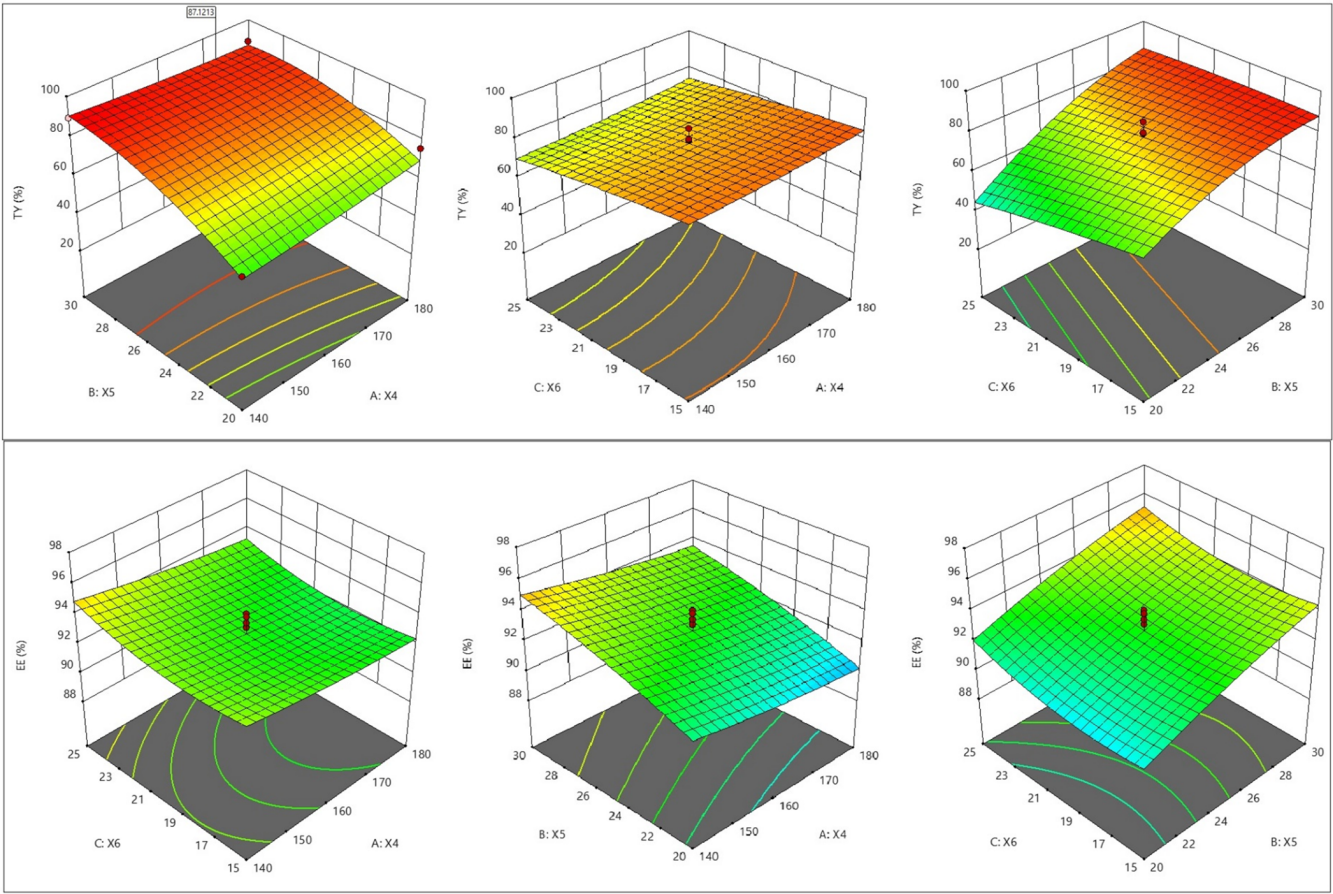
Total yield (%) (upper row) and encapsulation efficiency (%) (lower row) plots of the powders.

EE is the percentage of encapsulated oil in the total oil, and it is one of the important quality parameters in encapsulation of oils by spray drying. The highest microencapsulation efficiency (96.5%) was observed in run no. 11 and the lowest one (88.8%) was observed in run no. 19. With the decrease in the rate of airflow there was a decrease in EE (Figure 3). This may be due to the change in the airflow rate and solid content. Frascareli et al.^33^ investigated effect of process conditions on the microencapsulation of coffee oil by spray drying. They indicated that the solid content had a positive effect on the EE, and this result can be attributed to the emulsion droplet size, which decreased when the total solid content increased. Moreover Jafari et al.,^34^ indicated that lower emulsion droplet size leads to higher encapsulation efficiency of oils and flavours.

The coefficient of determination was determined as 0.754, indicating that 75% of total variability of the EE could be explained by the defined model. The ANOVA of quadratic model states that the model is significant (*p* < 0.05) with lack-of-fit value of 0.746. The actual equation of the quadratic model generated from the obtained data to predict the EE of powder in terms of coded variables is given in eq 4:
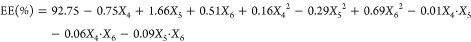
4where *X*_4_ is inlet temperature, *X*_5_ is air flow rate, and *X*_6_ is pump rate.

## 3 Conclusion

In this study, the optimization of microencapsulation conditions for EO by using response surface methodology (RSM) has been demonstrated. The RSM with five-level, three-factor CCC was employed with wall materials (ratio of MD:MS), oil concentration, and homogenization speed as independent variables, and their effects on emulsion characteristics were assessed. On the other hand, RSM with five-level, three-factor CCC was employed with inlet temperature, airflow rate, and pump rate as independent variables for encapsulation where their effects on total yield and EE were analyzed. According to the results, the homogenization speed was found to be the most significant factor (*p* < 0.05) affecting the emulsion properties. Each selected factor for spray drying (inlet temperature, air flow rate and pump rate) had a significant effect on the characteristics of obtained powders. Further studies are required to determine the oxidation stability of the optimized powder as well as its possible applications in food formulations.

## 4 Materials and Methods

### 4.1 Materials

All chemicals of analytical grade were purchased from Sigma-Aldrich, Germany. Echium oil (*Echium plantagineum* L.) was supplied from De Wit Specialty Oils, The Netherlands. Modified starch (MS) (EmCap) and maltodextrin (MD) (C*Dry MD 01915) were supplied from Cargill Co. (Istanbul, Türkiye Submission).

### 4.2 Methods

#### 4.2.1 Quality Properties and Fatty Acid Composition of EO

The acid and peroxide values of EO were determined according to the AOAC official standard methods (Method 922.11 and Method 965.33).^35,36^

The fatty acid composition of the oil was determined using the procedures described by Zahran and Tawfeuk.^37^ According to the method, the fatty acid composition was determined by the conversion of oil to fatty acid methyl esters prepared by adding 1.0 mL of *n*-hexane to 15 mg of oil, followed by the addition of 1.0 mL of sodium methoxide (0.4 mol). The mixtures were vortexed for 30 s and allowed to settle for 15 min. The upper phase containing the free fatty acid methyl esters of the oil was prepared and analyzed for its constituents by GLC. HP 6890 gas chromatograph occupied with a flame ionization detector (GC-FID, Hewlett-Packard, USA) was used for this purpose. Supelco SP-2380 (60 m × 0.25 mm × 0.20 μm) (Sigma-Aldrich, USA) capillary column was used for the analysis. The injector and detector temperatures were held at 250 °C. The oven temperature was initially held at 150 °C for 3 min, and temperature increased at a rate of 10 °C/min until 225 °C and held for 10 min. Helium was used as the carrier gas at a flow rate of 1.2 mL/min. A 1 μL of sample was injected into the GLC. Fatty acids were identified by retention times of standards of fatty acid methyl ester mix (Supelco, Germany) and expressed as a percentage of total peak area of all fatty acids in oil samples.^20^

#### 4.2.2 Experimental Design for RSM

RSM was applied to obtain the optimum conditions of emulsification and encapsulation of EO. Three factors and five levels CCC was used to optimize both emulsion preparation and spray drying conditions.^38^ The levels of the chosen factors are listed in Table 4 and Table 5. A total of 20 experimental runs with 5 center points were generated.

**Table 4:** Independent Variables and Their Ranges for Optimizing the Emulsification of EO

	Symbols		Levels	
Independent variables	Uncoded	Coded	Uncoded	Coded
Wall materials (%, w/w)	*W*	*X*_1_	93.4	2
			90	1
			85	0
			80	–1
			76.6	–2
Oil concentration (%, w/w)	*O*	*X*_2_	28.4	2
			25	1
			20	0
			15	–1
			11.6	–2
Homogenization speed (*x* × 1000 rpm)	*H*	*X*_3_	18.4	2
			15	1
			10	0
			5	–1
			1.6	–2

**Table 5:** Independent Variables and Their Ranges for Optimizing the Encapsulation of EO by the Spray Drying Method

	Symbols		Levels	
Independent variables	Uncoded	Coded	Uncoded	Coded
Inlet temperature (°C)	*T*_i_	*X*_1_	193.63	2
			160	1
			150	0
			140	–1
			126.36	–2
Air flow rate (%)	*A*	*X*_2_	33.4	2
			30	1
			25	0
			20	–1
Pump rate (%)	*P*	*X*_3_	16.6	–2
			28.4	2
			25	1
			20	0
			15	–1
			11.6	–2

#### 4.2.3 Emulsion Preparation

Three factors and their ranges chosen for emulsion preparation were ratio of wall materials (MD:MS) (X_1,%_) (80–90% w/w), oil concentration (X_2,%_) (15–25%, w/w), and homogenization speed (X_3_, rpm) (5–15 × 1000 rpm). Table 4 shows the emulsion conditions in these ranges. Based on the selected independent variables, RSM was used to investigate the effects of these factors on emulsion droplet size (μm) and viscosity (Pa·s).

The oil-in-water type emulsion consisted of a solution of MD and MS as the aqueous phase and EO as the oil phase. After preparation of the solutions of wall materials (with concentrations of 30% “w/w on wet basis” and 70% distilled water), they were stirred with a magnetic stirrer at room temperature (25 °C) to confirm a full saturation. Coarse emulsions were prepared by blending EO and the prepared wall solution using a T18 digital ULTRA-TURRAX homogenizer (IKA, Germany) for 5 min.^23^ The EO concentrations in the emulsion were 15, 20, and 25% of the total solids in the wall material solutions.

#### 4.2.4 Emulsion Characterization

##### 4.2.4.1 Emulsion Viscosity

The viscosity was determined by the steady-shear flow curve determination according to the method described by Carneiro et al. (2013), using a HAAKE RheoStress rheometer (Thermo-Fisher Scientific Inc. Waltham, USA). The measurements were performed in triplicate using plate–plate geometry of stainless steel with a diameter of 75 mm and a distance of 0.2 mm at 25 °C.

##### 4.2.4.2 Emulsion Droplet Size

The distribution of droplets was performed according to Giroux et al.^39^ (2010), by using a laser light diffraction instrument, Mastersizer S (Malvern Instruments, Malvern, UK). The emulsion droplet size was expressed as D_32_, the Sauter mean diameter.

##### 4.2.4.3 Optical Microscopy

Before analysis, emulsions were moderately homogenized in a glass tube. On a glass microscope slide, a drop of each emulsion was placed and then covered with a coverslip. Original emulsions were diluted with deionized water to 10% to obtain a better image. The microstructure of the emulsions was observed using an optical microscope (Nikon–Ni-U, CFI60 infinity optical system, Nikon Instruments Inc., Melville, USA) equipped with a CCD video camera module (microscope camera control unit DS-L4). The pictures were then acquired through a CCD camera-connected PC and a Digital Image Processing Software (version 6.0 of Image-Pro Plus).^40^

#### 4.2.5 Encapsulation

The spray drying process was performed using a laboratory scale spray dryer (Mini Spray Dryer B-290, BÜCHI Labortechnik AG, Flawil, Switzerland), occupied with a nozzle atomization system with a 1.5 mm diameter and 100% aspirator capacity. The emulsions were fed into the main chamber through a peristaltic pump, and the flow rate of the feed was controlled by the pump rotation speed.

Independent variables and their ranges for the spray drying process are inlet temperature (*X*_4_, °C) (140–180 °C), air flow rate (*X*_5_, %) (20–30%), and pump rate (*X*_6_, %) (15–25%). Note that, depending on the spray dryer model, air flow rate at 20 to 30% corresponds to 0.283 to 0.439 m^3^/h, respectively, and pump rate at 15 to 25% corresponds to 4.5–7.5 mL/min, respectively. In addition, the ranges of those values were determined according to the preliminary trials. Table 5 shows the spray drying conditions under these parameters.

Based on the selected independent variables such as inlet temperature, air flow rate, and pump rate of spray drying, RSM was used to investigate the effects of these factors on total yield (%) and encapsulation efficiency (%).

Coded and uncoded variables for emulsification and encapsulation operations are given in Table 4 and Table 5. Twenty experimental points were generated for each operating parameter with three factors and five levels by RSM using MODDE 13.0.1 (Umetrics, Sweden). The quadratic polynomial regression model was used for predicting responses.^38^

#### 4.2.6 Powder Characterization

##### 4.2.6.1 Encapsulation Efficiency

The amount of unencapsulated oil was measured using a method (i.e., surface oil) described by Tan, Chan and Heng.^41^ EE (%) was determined by using eq 5:
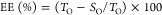
5where *T*_O_ is the total oil content and *S*_O_ is the surface (nonencapsulated) oil content.^34^

##### 4.2.6.2 Total Yield

Encapsulation yield based on dry matter content was calculated according to eq 6:^42^
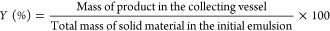
6

### 4.3 Statistical Analysis

Experimental design, data analysis, and response surface plots were prepared with Design-Expert 11 software (Stat-Ease, Inc., Minneapolis, MN, ABD). Second-order coefficients were generated by regression analysis whereas the goodness of fit of the models were evaluated by the coefficient of determination (R^2^) and ANOVA at 95% level of significance. A second-order model was used to fit the data according to model equation (eq 7) as follows:
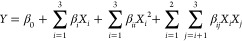
7where *Y* is the response, *β*_*0*_ is the intercept; *β*_*i*_ is the linear term (first-order model); *β*_*ii*_ is the quadratic term (second-order model), *β*_*ij*_ is the interaction regression coefficients, and *X*_*i*_ and *X*_*j*_ are the independent variables.^43^
